# Diagnosis of takotsubo cardiomyopathy is increasing over time in patients presenting as ST-elevation myocardial infarction

**DOI:** 10.1007/s12471-016-0859-x

**Published:** 2016-07-13

**Authors:** A.M. Otten, J.P. Ottervanger, T. Symersky, H. Suryapranata, M.J. de Boer, A.H.E.M. Maas

**Affiliations:** 1 Department of Cardiology, Isala Klinieken, Zwolle, The Netherlands; 2 Department of Cardiology, Radboud University Medical Center Nijmegen, Nijmegen, The Netherlands

**Keywords:** Takotsubo cardiomyopathy, Gender, STEMI

## Abstract

**Background:**

Takotsubo cardiomyopathy often presents with the clinical signs of ST-elevation myocardial infarction (STEMI). The increase in scientific publications addressing this relatively rare condition may result in higher awareness and diagnosis of takotsubo cardiomyopathy.

**Aim:**

To assess the observed prevalence per year of takotsubo cardiomyopathy in a large registry of patients with STEMI, during a 12-year inclusion period.

**Method:**

All patients presenting with STEMI at a large regional cardiology clinic were entered into a database (*n* = 8,413, mean age 63 ± 13 years). Takotsubo cardiomyopathy was diagnosed in 42 patients (0.5 %). Years of evaluation were defined as ‘early years’ (January 2002 to December 2007; *n* = 4350) and ‘later years’ (January 2008 to December 2013). Multivariable analyses were performed to adjust for differences in demographical and clinical variables.

**Results:**

In later years, the age of STEMI patients was slightly higher (64 ± 13 vs. 63 ± 13 years, *p* < 0.001), with more patients with clinical symptoms of shock (10 vs. 7 %, *p* < 0.001) or a history of percutaneous coronary intervention or hypertension (10 vs. 8 %, *p* = 0.001 and 37 vs. 34 %, *p* < 0.001). Smoking and a positive family history were less often observed during later years (39 vs. 46 %, *p* < 0.001 and 37 vs. 42 % *p* < 0.001). Patients with takotsubo cardiomyopathy were more often female (81 vs. 27 %, *p* = 0.001). Takotsubo cardiomyopathy was more often diagnosed in the later period (0.7 vs. 0.3 %, OR 2.4, 95 % CI 1.2–4.6, *p* = 0.009). The higher prevalence of takotsubo cardiomyopathy in recent years remained significant after adjustment for differences in patient characteristics (OR 2.1, 95 % CI 1.1–4.3).

**Conclusion:**

Takotsubo cardiomyopathy is currently more often diagnosed in patients with STEMI compared with in earlier years. This is probably due to the increased scientific and clinical awareness among doctors, but the prevalence is still low.

## Introduction

Takotsubo cardiomyopathy is characterised by transient wall motion abnormalities mimicking ST-elevation myocardial infarction (STEMI). It was first described in Japan in 1991 [1]. At that time, takotsubo cardiomyopathy was completely unrecognised in Europe and North America and it was thought to only occur in Asia, where the first cohorts were published approximately 10 years later [2, 3]. The first observation of takotsubo cardiomyopathy in Caucasian patients was published in 2003 [4]. Since then, awareness of takotsubo cardiomyopathy among cardiologists in Europe and North America has increased, with more patients initially presenting with STEMI being diagnosed with takotsubo cardiomyopathy. However, it is also likely that a distinctly different population of patients are referred for STEMI over time due to improved therapy, logistics and modifications in referral [5, 6]. This may contribute to a shift in the observed numbers of takotsubo cardiomyopathy. For instance, as currently more elderly women with STEMI are referred for immediate percutaneous coronary intervention (PCI), the number of observed cases of takotsubo cardiomyopathy may have increased, since it is more prevalent in postmenopausal women [7, 8]. To assess alterations in the observed prevalence of takotsubo cardiomyopathy in patients with STEMI, adjustments should be made for these potential confounding factors [3, 9, 10]. In the current study, we investigated whether the observed number of patients with takotsubo cardiomyopathy changed over a 12-year period and if this is related to alterations in patient characteristics.

## Methods

From January 2002 to December 2013, individual data from all STEMI patients who were considered for primary PCI and who underwent early coronary angiography at our centre, were prospectively recorded in a dedicated database. Early years were defined as January 2002 until December 2007 and later years as January 2008 until December 2013. Patients were diagnosed with STEMI if they had chest pain lasting longer than 30 minutes and ECG changes with ST elevation >2 mm in at least two precordial leads or >1 mm in the limb leads. Cardiac biomarkers were elevated in all patients. Information on demographic variables was directly registered at first contact with the patient, including age, sex, medical history, family history and traditional cardiovascular risk factors. In patients in whom echocardiography was performed during admission or after one month to assess LV function, the echocardiogram was reviewed by an expert cardiologist or a resident in cardiology supervised by an expert. In order to investigate the number of publications concerning takotsubo cardiomyopathy, we sought in the PubMed database with the following search strategy per year: (“2002/01/01” [Date-Publication] : “2002/12/31” [Date-Publication]) AND ((tako AND tsubo) OR takotsubo). Although takotsubo cardiomyopathy is sometimes mentioned as ‘ampulla cardiomyopathy’, ‘apical ballooning syndrome’, ‘broken heart’ or ‘stress-induced cardiomyopathy’ in the literature, almost all articles were still revealed with the search strategy because takotsubo or tako tsubo was mentioned in the article [11–13].

## Definition of takotsubo

ECG changes (either ST-segment elevation and/or T‑wave inversion) are major diagnostic criteria for takotsubo cardiomyopathy. Since all patients in our database had ST elevation, this criterion was fulfilled for all patients. The presence of an epicardial stenosis or spasm of a coronary artery perfusing the territory of hypokinesia or akinesia of the myocardium are exclusion criteria for takotsubo cardiomyopathy [7, 14]. Patients with takotsubo cardiomyopathy had transient hypokinesia, dyskinesia or akinesia of the LV mid segments with or without apical involvement according to the diagnostic criteria of the Mayo Clinic [10].

## Statistical analysis

Statistical analysis was performed using SPSS version 20 (SPSS Inc, Chicago, IL). Continuous data were expressed as mean and standard deviation and categorical data as percentages. Tests for significance were two-sided and values with an α of 0.05 were considered significant. In order to analyse whether an independent association was present for the number of patients observed with takotsubo cardiomyopathy over time and the number of publications, we used binary logistic regression comparing early years with later years. The multivariate model consisted of all baseline variables with a *p* ≤ 0.1 (gender, age, previous PCI, hypertension, smoking, hypercholesterolaemia, positive family history and Killip class ≥2).

## Results

In total, 8,413 patients with a STEMI were referred to our centre during a 12-year time period. The mean age was 63 ± 13 years. During the study period, 685 (8 %) patients had no identifiable stenosis or spasm of a coronary artery at angiography. These patients may have had takotsubo cardiomyopathy according to the Mayo Clinic’s diagnostic criteria [10]. All patients had a complete resolution of the myocardial segments on echocardiography or angiography within a month. Two patients with myocarditis were excluded and there were no patients with pheochromocytoma. Of these 685 patients, one month follow-up data on left ventricular function were missing in two patients from 2002, therefore they were excluded from further analysis. In total, 42 patients (0.5 %) were diagnosed with takotsubo cardiomyopathy according to the criteria as described in the methods.

The 643 patients who were treated conservatively and did not have takotsubo cardiomyopathy were older than invasively treated patients (65 ± 16 vs. 63 ± 12 years, *p* = 0.002). They were also more often female (31 vs. 27 %, *p* = 0.01), more often had a history of myocardial infarction (15 vs. 10 %, *p* < 0.001), a history of coronary artery bypass graft (9 vs. 3 %, *p* < 0.001), a history of PCI (12 vs. 9 %, *p* = 0.02), a history of cerebrovascular accident (6 vs. 3 %, *p* < 0.001), hypertension (41 vs. 36 %, *p* = 0.007) and diabetes (14 vs. 11 %, *p* = 0.03). Patients treated conservatively less often had a positive family history (31 vs. 40 %, *p* < 0.001) and were less often smokers (30 vs. 44 %, *p* < 0.001).

## Trends over the years in patients referred for STEMI

Compared with earlier years, patients referred for STEMI between 2008–2013 were older and more often had a history of previous PCI, hypertension, and a higher Killip class at admission. Current smoking and a positive family history were less often present in later years. Furthermore, the duration of hospital stay was slightly longer in the later period. The other clinical characteristics including gender did not change over time (Tab. 1).

**Tab. 1:** Clinical characteristics of patients referred for ST-elevation myocardial infarction in early (2002–2008) and later (2008–2013) years

		Early years (*n* = 4,350)	Later years (*n* = 4,063)	P-value
Age		63 ± 13	64 ± 13	0.001
Gender (women)		1,165 (27 %)	1,154 (28 %)	0.10
Observed takotsubo cardiomyopathy		13 (0.3 %)	27 (0.7 %)	0.007
BMI (kg/m^2^)		27 ± 5	28 ± 8	0.20
History of	MI	441 (10 %)	412 (10 %)	0.88
	CABG	156 (4 %)	135 (3 %)	0.56
	PCI	356 (8 %)	414 (10 %)	0.001
	Stroke	129 (3 %)	134 (3 %)	0.36
	Diabetes	508 (12 %)	458 (11 %)	0.59
	Hypertension	1,454 (34 %)	1,557 (37 %)	<0.001
Positive family history		1,710 (42 %)	1,413 (37 %)	<0.001
Smoking (ever)		1,925 (46 %)	1,557 (39 %)	<0.001
Hypercholesterolaemia		958 (23 %)	840 (22 %)	0.07
Killip class >2 on admission		302 (7 %)	283 (10 %)	<0.001
Hospital stay (days)		4 ± 6	5 ± 8	<0.001
Heart frequency (min)		76 ± 18	76 ± 19	0.023
Systolic BP at admission (mmHg)		133 ± 25	132 ± 27	0.70
Diastolic BP at admission (mmHg)		80 ± 16	80 ± 23	0.78

*BMI* body mass index, *BP* blood pressure, *CABG* coronary artery bypass graft, *MI* myocardial infarction, *PCI* percutaneous coronary intervention

## Difference in patients with and without takotsubo

Patients with takotsubo cardiomyopathy were more often older, female, less often current smokers and had a higher heart rate at admission compared with the usual STEMI patients (Tab. 2). All other cardiovascular risk factors and haemodynamic parameters were comparable between patients with and without takotsubo cardiomyopathy.

**Tab. 2:** Comparison of clinical characteristics between patients with and without takotsubo cardiomyopathy in 8,413 patients admitted with ST-elevation myocardial infarction

		Patients without takotsubo cardiomyopathy (*n* = 8,371)	Patients with takotsubo cardiomyopathy (*n* = 42)	*p*-value
Age		63 ± 13	66 ± 13	0.13
Gender (women)		2,285 (27 %)	34 (81 %)	<0.001
BMI (kg/m^2^)		28 ± 13	25 ± 4	0.22
History of	MI	850 (10 %)	3 (7 %)	0.54
	CABG	291 (4 %)	0 (0 %)	0.23
	PCI	769 (9 %)	1 (2 %)	0.13
	Stroke	261 (3 %)	2 (5 %)	0.53
	Diabetes	963 (12 %)	3 (7 %)	0.40
	Hypertension	2,999 (36 %)	12 (29 %)	0.35
Positive family history		3,113 (39 %)	10 (25 %)	0.08
Smoking (ever)		3,477 (43 %)	5 (12 %)	<0.001
Hypercholesterolaemia		1,792 (22 %)	6 (15 %)	0.24
Killip class ≥2 on admission		582 (8 %)	3 (9 %)	0.80
Hospital stay (days)		4 ± 7	3 ± 3	0.21
Heart rate (min)		76 ± 21	83 ± 15	0.04
Systolic BP on admission (mmHg)		133 ± 27	135 ± 29	0.47
Diastolic BP on admission (mmHg)		76 ± 19	80 ± 15	0.90

*BMI* body mass index, *BP* blood pressure, *CABG* coronary artery bypass graft, *MI* myocardial infarction, *PCI* percutaneous coronary intervention

## Trends in prevalence of takotsubo cardiomyopathy over the years

**Fig. 1:**
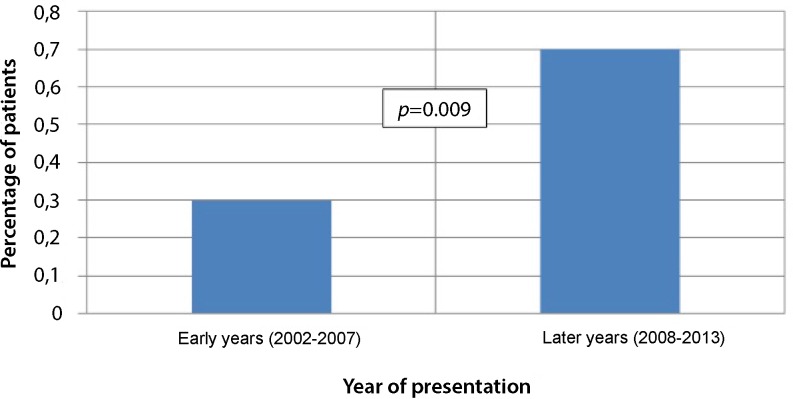
Percentage of observed takotsubo presentiegeld as STEMI

The annual observed prevalence of takotsubo cardiomyopathy in patients with STEMI ranged from 0 % in 2002 to 1.05 % in 2009 and increased significantly over time (Fig. 1). Compared with the number of patients with takotsubo cardiomyopathy in the early years, takotsubo cardiomyopathy was more often observed in the later period (OR 2.4, 95 % CI 1.2–4.6). In the multivariate model, this difference remained significant (OR 2.1, 95 % CI 1.1–4.3).

**Fig. 2:**
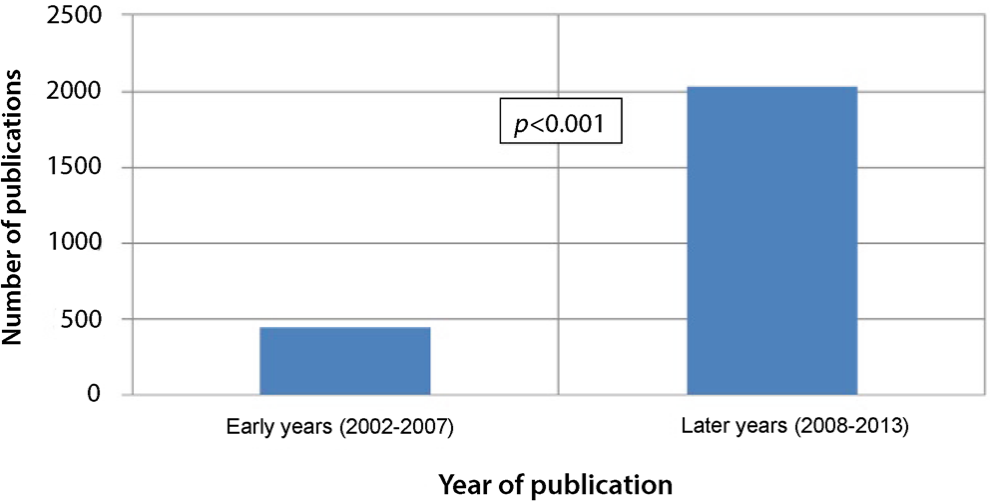
Number of publications in PubMed with takotsubo in the title

The literature search showed that in the early years, takotsubo was less often mentioned in the literature compared with later (Fig. 2).

## Discussion

In this large cohort of patients with STEMI, we found a significant increase in the number of patients observed with takotsubo cardiomyopathy over time, independent of patient characteristics. However, the prevalence of takotsubo cardiomyopathy among STEMI patients is still low.

The prevalence of takotsubo cardiomyopathy in the literature ranges from 0.02 % in the general population [14] to 1–2 % in populations with acute coronary syndromes [7]. However, these prevalences cannot be compared with our study because we present a cohort of patients with STEMI. To our knowledge, this is the first STEMI population to be studied for takotsubo cardiomyopathy.

The increased number of observed takotsubo cardiomyopathy patients over the years may have different explanations. Firstly, since patients with STEMI are currently older and takotsubo cardiomyopathy is associated with increasing age, takotsubo cardiomyopathy may be more often diagnosed [15–17]. However, this cannot be the entire explanation for the increased number of observed cases of takotsubo cardiomyopathy, because after correcting for age in the multivariate model there was still a relationship between later years and increase of takotsubo cardiomyopathy in our study. Another explanation for this increase may be changed risk profiles of admitted STEMI patients over the years, which was also observed in other studies [18, 19]. These changes in risk factors only partly explain the difference in observed frequency of takotsubo cardiomyopathy in our study, since after multivariable analyses takotsubo cardiomyopathy was still more prevalent in recent years. A third and probably most important reason for the increased prevalence may be the improved network and facilities in the treatment of acute STEMI patients. In fact, almost all STEMI patients are currently referred to a tertiary centre with primary PCI facilities. Furthermore, improved recognition of takotsubo cardiomyopathy may also play an important role, which may be related to a higher awareness of cardiologists and other medical personnel due to an increasing number of scientific studies on takotsubo cardiomyopathy in the literature and communications at conferences (Fig. 2). Large systemic registries or multicentre trials [20–22] do not only increase awareness, they also result in a better understanding of the demographics and ultimately also the treatment of takotsubo cardiomyopathy. Furthermore, case reports concerning a new clinical presentation of takotsubo cardiomyopathy are more often published [23, 24].

In our study, LV function was assessed with an echo or LV angiogram primarily in the acute situation after STEMI. Therefore, we used only LV function assessment in the acute setting. However, standard evaluation of LV function both acutely and after one month can avoid misdiagnosis of takotsubo cardiomyopathy. The ESC guidelines also recommend evaluation of resting LV function both acutely and after more than two weeks after STEMI [25]. If the outpatient LV assessment is performed four weeks after STEMI, most takotsubo cardiomyopathy patients can be diagnosed with more certainty, because LV function due to takotsubo cardiomyopathy should be normalised within four weeks according to the definition of takotsubo cardiomyopathy [26].

Although we evaluated each STEMI patient without coronary intervention for possible takotsubo cardiomyopathy, only 0.5 % of all patients with STEMI admitted in our hospital were diagnosed as takotsubo cardiomyopathy. Our observed prevalence of takotsubo cardiomyopathy is lower than estimates in the literature and therefore it is likely that we may have underdiagnosed takotsubo cardiomyopathy in our database [9, 27, 28]. Particularly in patient groups with a high prevalence of coronary artery disease, takotsubo cardiomyopathy may be underdiagnosed. As both coronary artery disease and takotsubo cardiomyopathy might be present in these patients and takotsubo cardiomyopathy can only be diagnosed in the absence of a significant coronary artery stenosis, takotsubo cardiomyopathy may not be recognised [27].

It is important to discriminate takotsubo cardiomyopathy with heart failure from STEMI with heart failure because it may have major implications for treatment.

Firstly, although it has not been properly studied, medical treatment for systolic heart failure can be considered in takotsubo cardiomyopathy patients in order to relieve acute symptoms (e. g. oedema) [29]. Three important drugs to use to these patients are mineralocorticoid receptor antagonists, beta blockers and angiotensin-converting enzyme (ACE) inhibitors (or angiotensin receptor blocker) [30]. When LV function normalises in patients with takotsubo cardiomyopathy after one month, it could be considered to stop the ACE inhibitor and mineralocorticoid-receptor antagonist to avoid adverse events associated with these drugs [31]. Although evidence is limited, the continuation of beta blockers can be considered in patients with takotsubo cardiomyopathy, because of the theory that takotsubo cardiomyopathy is caused by sympathetic hyperactivity [32].

Secondly, temporal aggressive heart failure treatment as LV assist devices may be considered in takotsubo cardiomyopathy patients if needed, even if the patient is not a candidate for heart transplant [33]. Misdiagnosing takotsubo cardiomyopathy for type 1 myocardial infarction may result in an unnecessary, long-term treatment with dual antiplatelet therapy. Without plaque rupture, only long-term prescription of acetylsalicylic acid is indicated and without coronary artery disease no antiplatelet therapy at all should be given in takotsubo cardiomyopathy patients. This will potentially diminish bleeding complications [34]. Finally, since takotsubo cardiomyopathy has a good prognosis in most patients, especially after four weeks, when the LV function normalises, patients may receive more adequate information about their prognosis.

There are several opportunities to improve the diagnosis of takotsubo cardiomyopathy. First of all, increased awareness in doctors and (possibly) patients is necessary. Moreover, a history of emotional trigger(s) must prompt the doctor to include takotsubo cardiomyopathy in the differential diagnosis. This is especially so in elderly women presenting with STEMI, because the prevalence of takotsubo cardiomyopathy is highest in this patient group [10]. The evaluation of possible emotional triggers should be done routinely in every patient with STEMI. Furthermore, when coronary stenosis but not coronary occlusion is observed with invasive angiography, fractional flow reserve could be considered in a later phase to evaluate whether the stenosis is haemodynamically significant. If no epicardial coronary stenosis is found as the cause of the STEMI at coronary angiography, apart from routine echocardiography, LV angiography can visualise an apical ballooning pattern of the left ventricle.

### Study limitations

This study was performed retrospectively in the first years and not all patients received sequential imaging of LV function. Therefore, it is likely that especially patients with takotsubo cardiomyopathy were underdiagnosed.

## Conclusions

We observed an increased frequency of takotsubo cardiomyopathy over the years. Since this difference remained irrespective of classical cardiovascular risk factors, and since takotsubo cardiomyopathy received more attention in the literature in the course of this study, it is likely that raised awareness contributed to this increase. National registries for patients with takotsubo cardiomyopathy may further increase awareness and stimulate scientific research about aetiology and (medical) treatment of takotsubo cardiomyopathy.
